# Metastatic HER2+ Breast Cancer: A Potentially Curable Disease?

**DOI:** 10.7759/cureus.1654

**Published:** 2017-09-05

**Authors:** Lisa Prior, Marvin Lim, Cian Ward, Hannah Featherstone, Hazel Murray, Clare D'Arcy, John Crown, Giuseppe Gullo

**Affiliations:** 1 Department of Medical Oncology, St Vincent's University Hospital, Dublin, Ireland; 2 Pathology, St Vincent's University Hospital, Dublin, Ireland

**Keywords:** metastatic, her2, durable complete remission, trastuzumab, breast cancer

## Abstract

The advent of trastuzumab and other human epidermal growth factor receptor 2 (HER2)-directed therapies has revolutionized the treatment of metastatic HER2-positive breast cancer, leading to prolonged survival and appreciable clinical benefit for a substantial subset of patients. Previously, in a retrospective study at our institution, we observed that nearly 10% of patients achieved a durable complete remission (DCR) following a combination of HER2-directed therapy and cytotoxic chemotherapy. We are currently expanding this study to include patients who were treated since the initial introduction of trastuzumab. From our ongoing study, we present a selected case series of three patients with metastatic HER2-positive breast cancer who achieved a DCR. It is theorized that metastatic HER2-positive breast cancer may be potentially curable in certain patients with favorable clinicopathological and molecular factors, which the patients within our case series mostly demonstrate. These include de novo presentation, estrogen receptor (ER)-negative status, limited disease burden, and absence of deleterious gene or pathway mutations. More research is needed in order to incorporate these findings into clinical practice.

## Introduction

The introduction of trastuzumab and other human epidermal growth factor receptor 2 (HER2)-directed therapies in the late 1990s dramatically altered the natural history of metastatic HER2-positive breast cancer, transforming it from an aggressive cancer subtype with a poor prognosis to one in which prolonged survival was possible [1-2]. HER2 is a transmembrane glycoprotein epidermal growth factor receptor with tyrosine kinase activity. It is overexpressed in approximately 15% of breast cancers and its activation is associated with increased cell growth, survival, and possibly angiogenesis [3-4]. In 2012, our institution published the first reported retrospective series of long-term follow-up of patients with metastatic HER2-positive breast cancer who achieved complete remission (CR) following a combination of chemotherapy (including anthracyclines and taxanes) and trastuzumab. Astonishingly, nearly 10% of patients with metastatic HER2-positive breast cancer achieved a durable complete remission (DCR), defined as a CR based on Response Evaluation Criteria in Solid Tumors (RECIST) 1.1, lasting for at least 36 months [5]. We are currently expanding this study with patients who were diagnosed and treated since the first introduction of trastuzumab. We present here a selected case series of three patients treated at our institution who achieved a DCR following a combination of HER2-directed therapy and cytotoxic chemotherapy (Table 1). Following on from this, we examine the existing evidence that illustrates why certain patients may be exceptional responders.

**Table 1:** Demographic data table IDC: Invasive ductal carcinoma G: Grade ER: Estrogen receptor PR: Progesterone receptor IHC: Immunohistochemistry WLE: Wide local excision ICORG: All-Ireland Cooperative Oncology Research Group

Patient	1	2	3
Age (years)	49	73	61
Type of breast cancer	IDC, G2, ER-/PR- negative, HER2-positive on IHC 3+	IDC, G3, ER-/PR-negative, HER2-positive on IHC 3+	IDC, G2-3, ER-/PR- negative, HER2-positive on IHC 3+
Stage at diagnosis	Stage 4 with liver, lung, and lymph node metastases	Stage 4 with lung and lymph node metastases	Stage 4 with liver and lymph node metastases
Treatment regimen	Paclitaxel and Herceptin (ICORG 11-10 trial) with Pertuzumab added on at later stage	Docetaxel, lapatinib, and Herceptin (EGF100-161 trial)	Docetaxel, lapatinib, and Herceptin (EGF100-161 trial) Liver metastasis resection and WLE of primary breast mass
Period of remission (years)	4.5	8	8

## Case presentation

Patient 1

**Figure 1:**
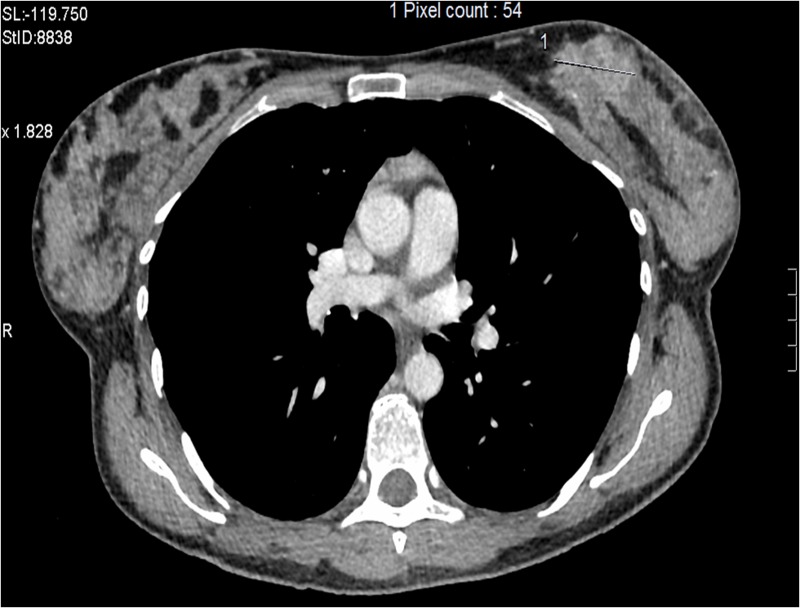
Patient 1: Baseline computed tomography (CT) at diagnosis reveals left breast mass

**Figure 2:**
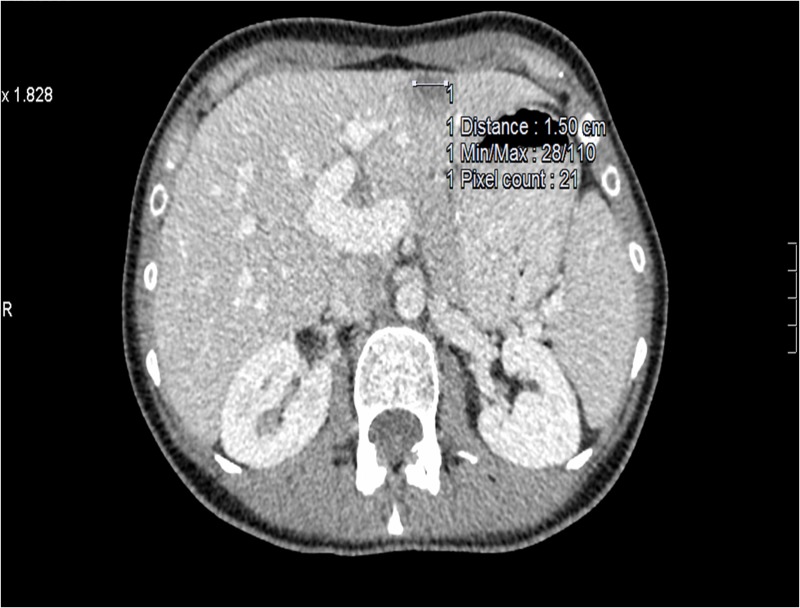
Patient 1: Baseline computed tomography (CT) at diagnosis demonstrates single liver lesion

A 49-year-old female was diagnosed with de novo metastatic HER2-positive/ER-negative and progesterone receptor (PR)-negative breast cancer in December 2012. Initial staging revealed a primary left breast tumor (Figure 1), ipsilateral axillary lymphadenopathy, multiple suspicious small lung nodules, and a solitary liver metastasis (Figure 2).

**Figure 3:**
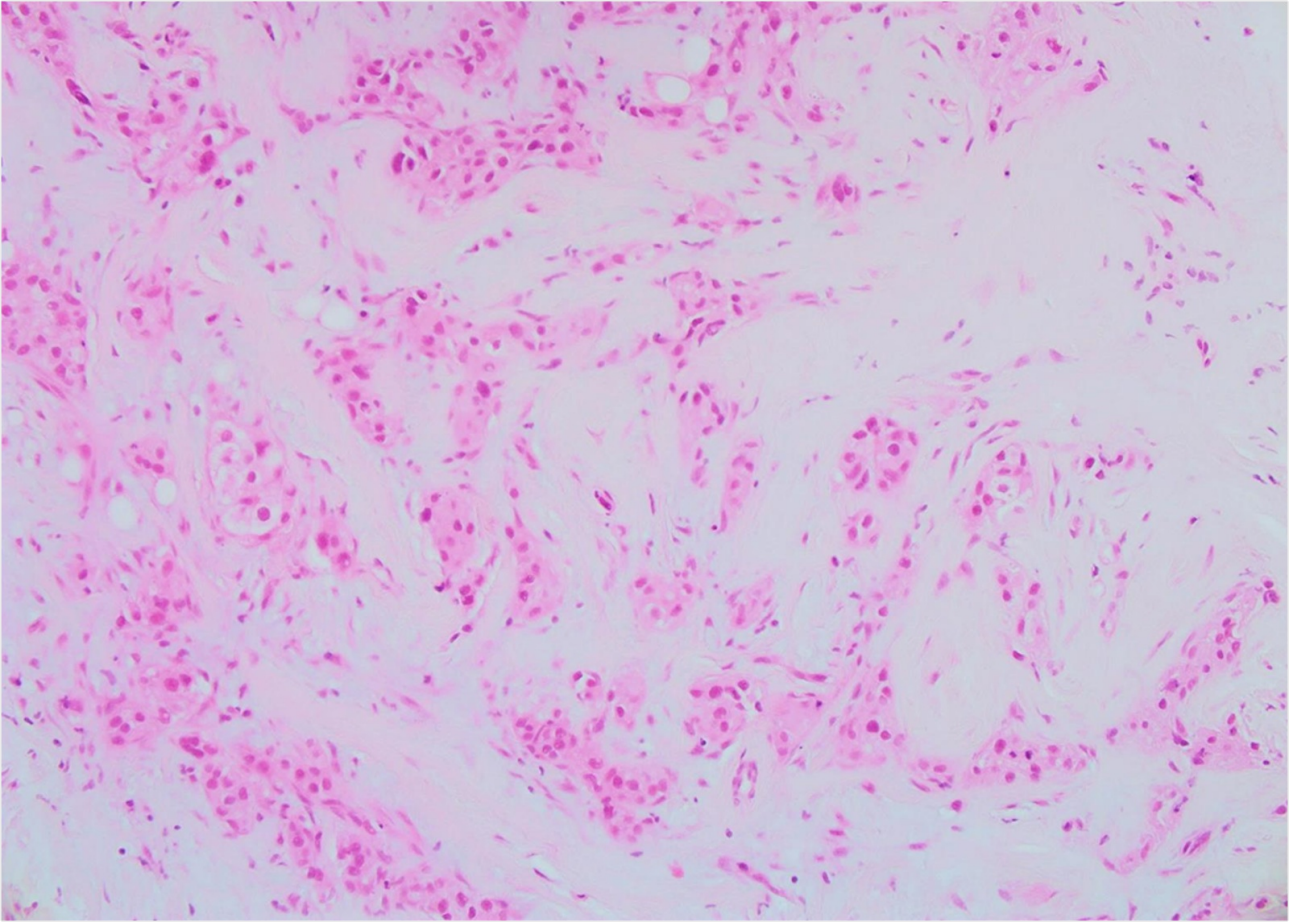
Patient 1: Biopsy of left breast tissue infiltrated by invasive ductal carcinoma (IDC) with grade 2–3 features (20X)

**Figure 4:**
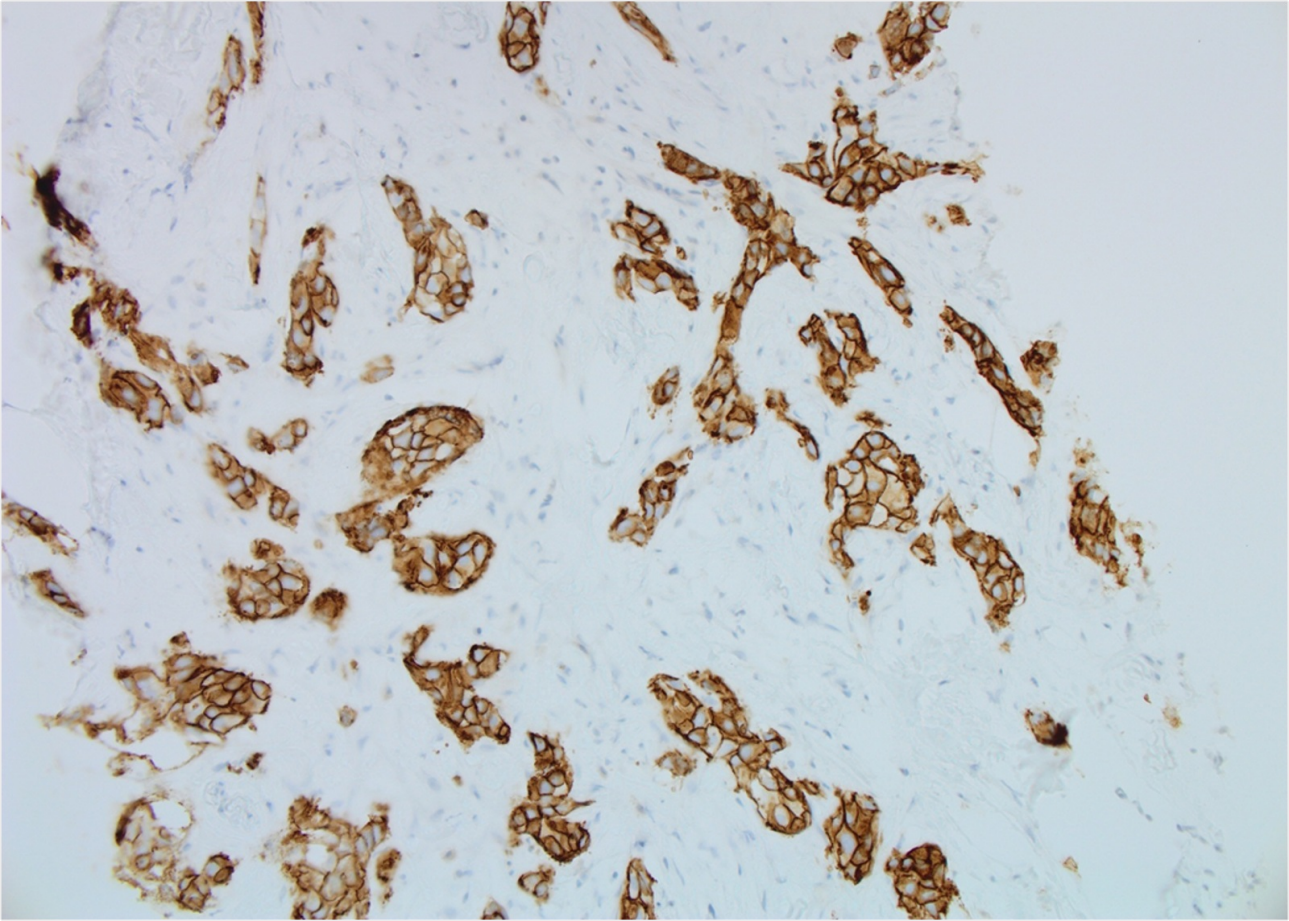
Patient 1: Tumor cells show strong membranous positivity for human epidermal growth factor receptor 2 (HER2) (score 3+) (20X)

**Figure 5:**
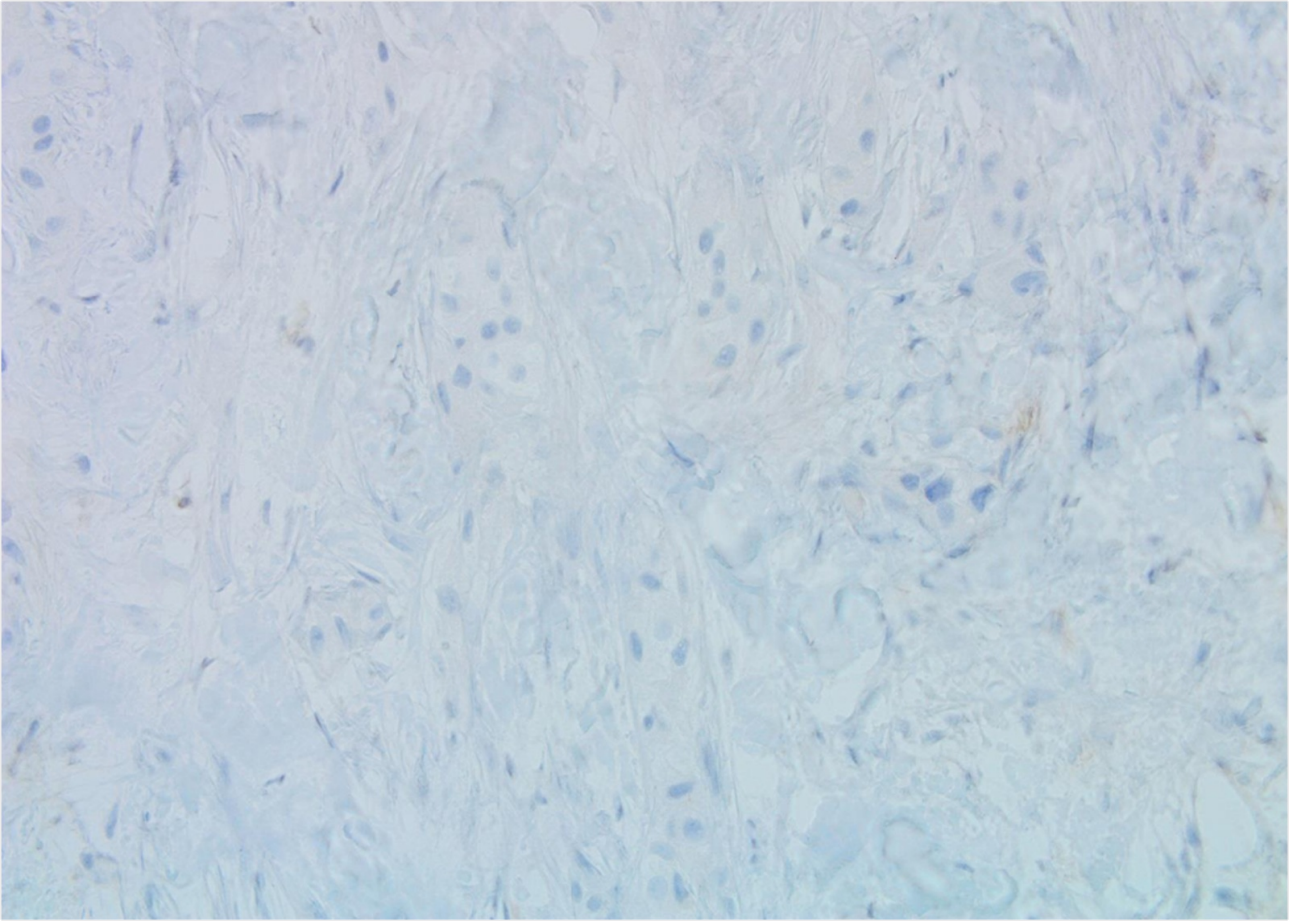
Patient 1: Tumour cells are estrogen receptor (ER)-negative (40X)

**Figure 6:**
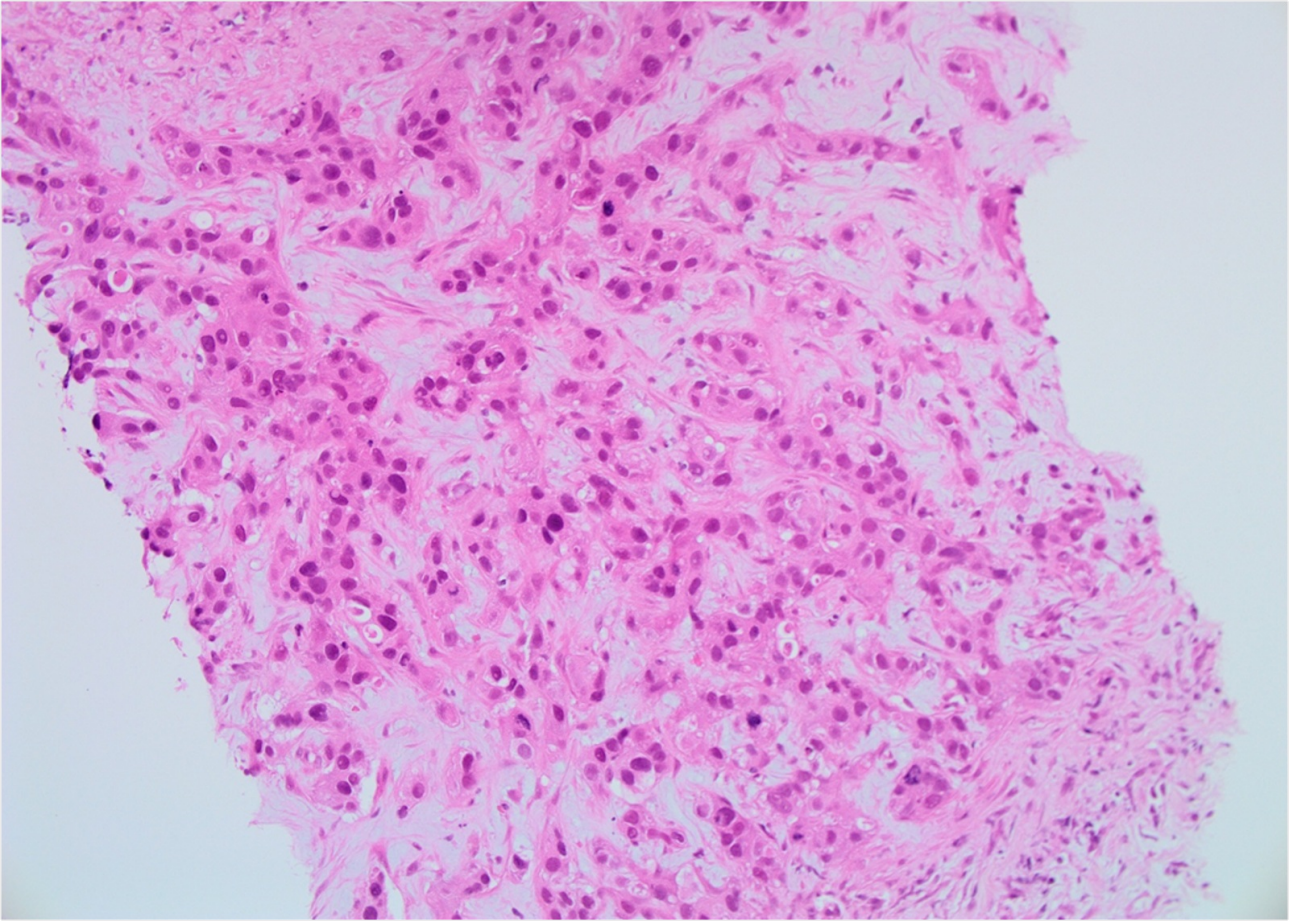
Patient 1: Biopsy of liver tissue infiltrated by metastatic ductal carcinoma (20X)

Both core breast and liver biopsies (Figures 3-6) confirmed a grade-II invasive ductal carcinoma (IDC) that was ER- and PR-negative and strongly HER2-positive (3+ on immunohistochemistry). She was commenced on weekly paclitaxel and trastuzumab in the context of a clinical trial in December 2012, which she tolerated well with minimal toxicities.

**Figure 7:**
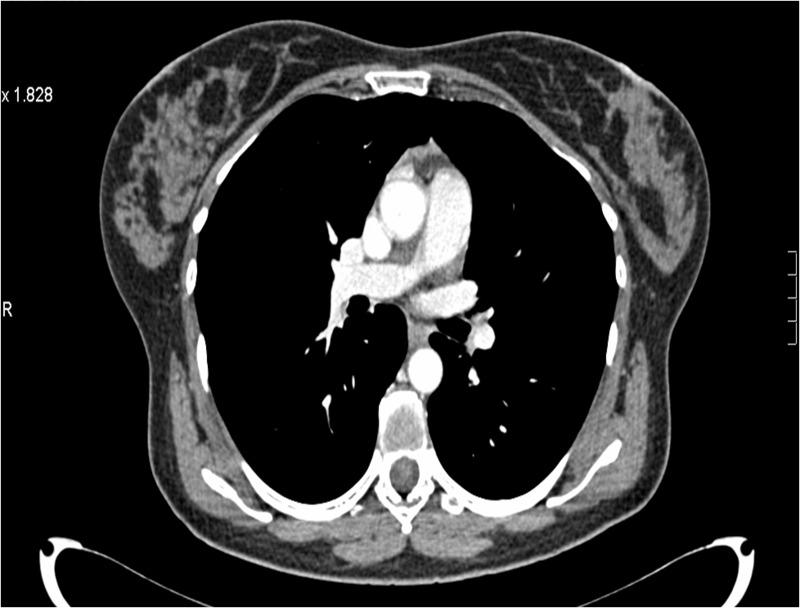
Patient 1: Post-treatment computed tomography (CT) shows resolution of left breast mass

**Figure 8:**
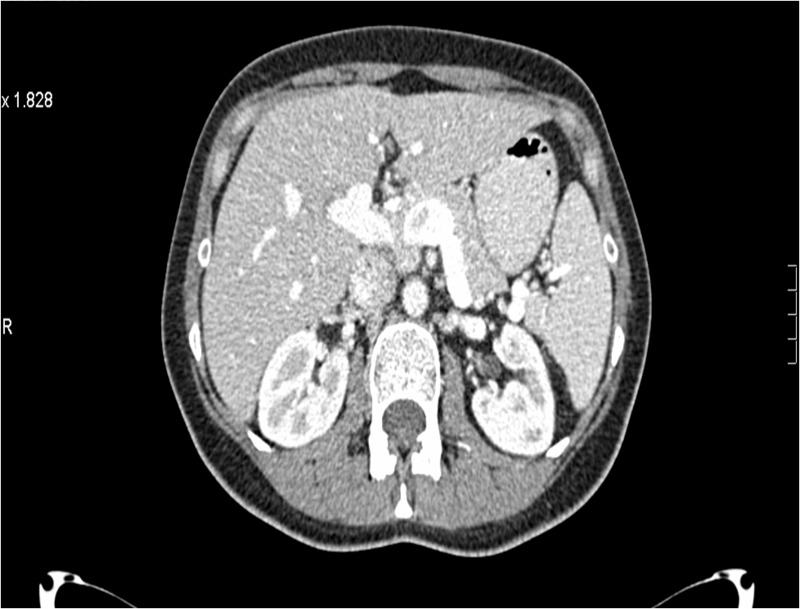
Patient 1: Post-treatment CT shows resolution of liver lesion

After eight months on treatment, she was confirmed on imaging to have achieved a CR to therapy (Figures 7-8). Systemic chemotherapy was discontinued in November 2014 after approximately 23 months and she continued on maintenance three-weekly single-agent trastuzumab. Pertuzumab was added to trastuzumab in October 2015 because of emerging evidence at the time of the survival benefit of dual HER2-inhibition in the maintenance phase. She currently remains in CR 4.5 years after diagnosis.

Patient 2

**Figure 9:**
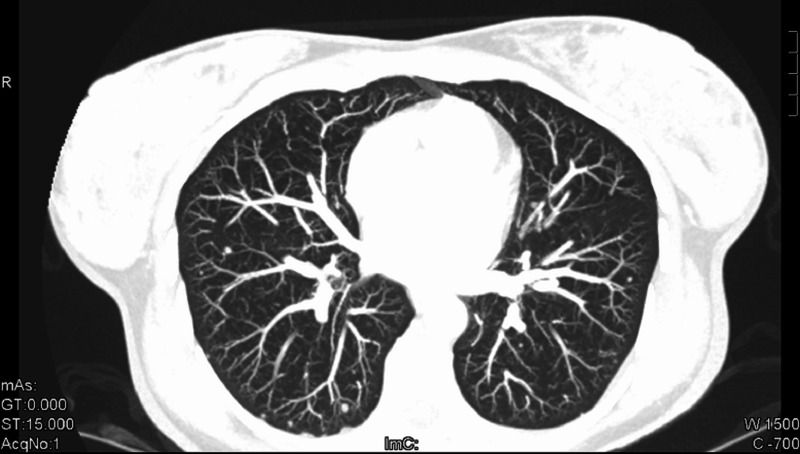
Patient 2: CT imaging demonstrates lung nodules pre treatment

**Figure 10:**
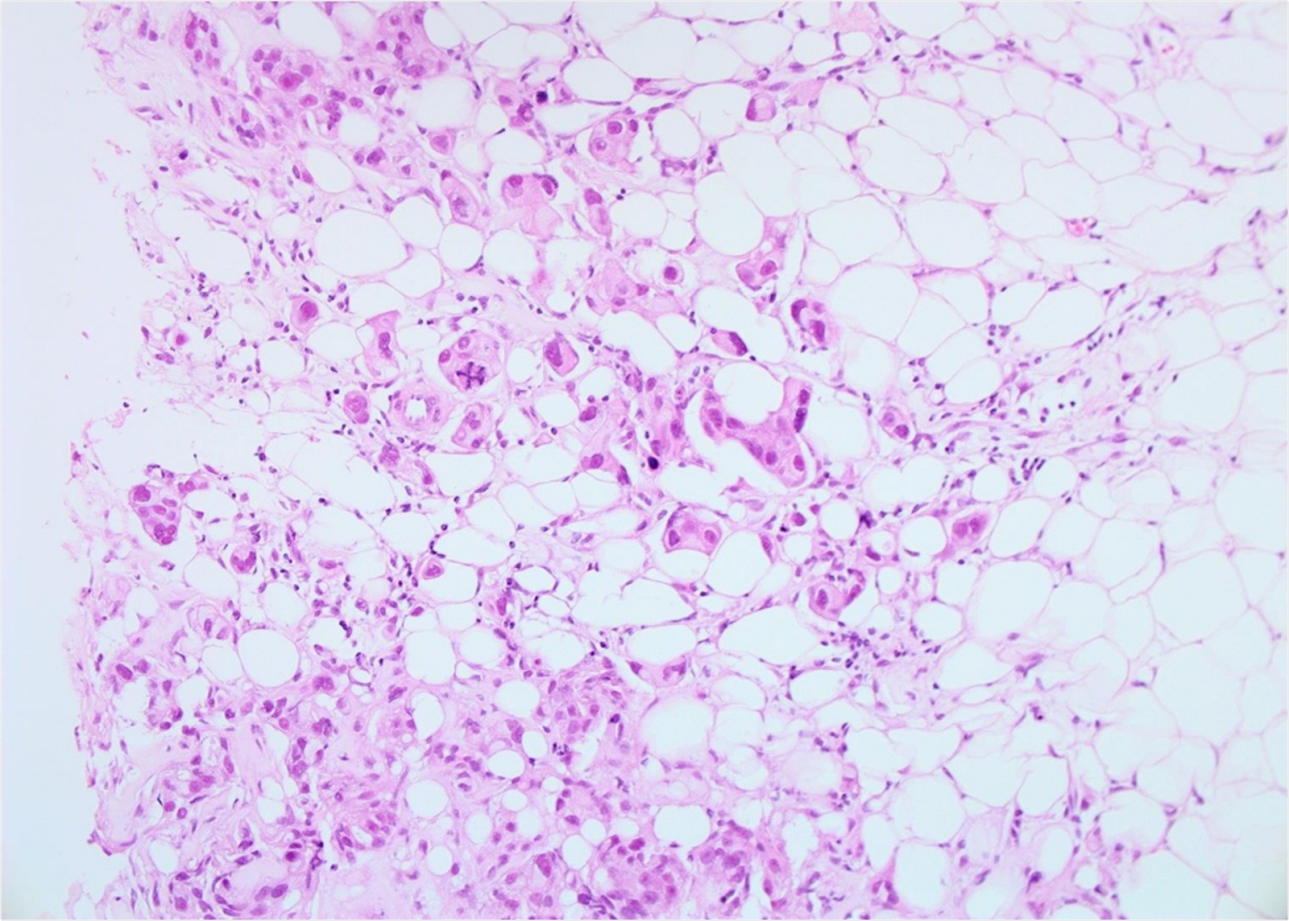
Patient 2: Biopsy of right breast tissue infiltrated by invasive ductal carcinoma with grade 3 features (20 X)

**Figure 11:**
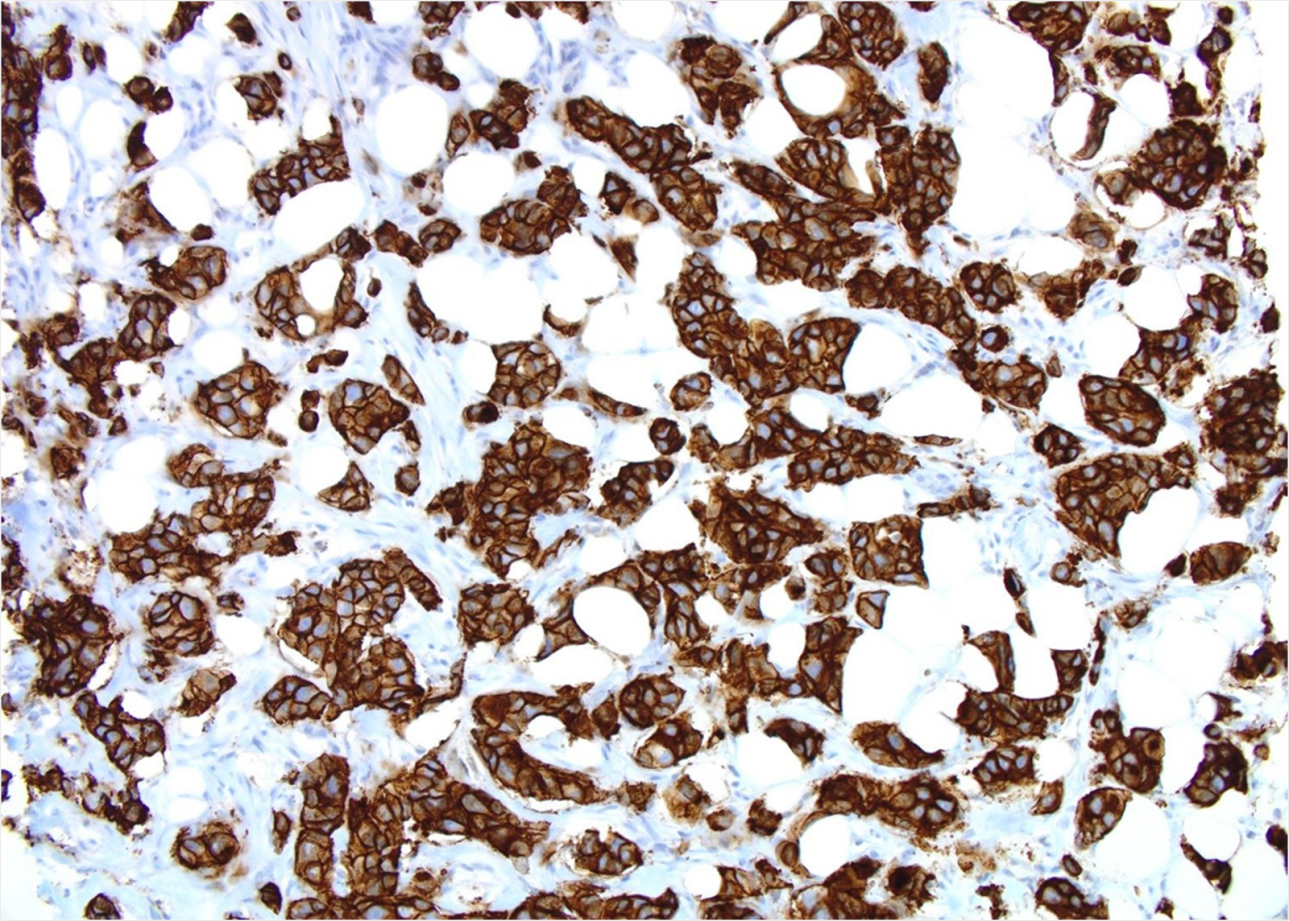
Patient 2: Tumor cells show strong membranous positivity for HER2 (score 3+) (20X)

**Figure 12:**
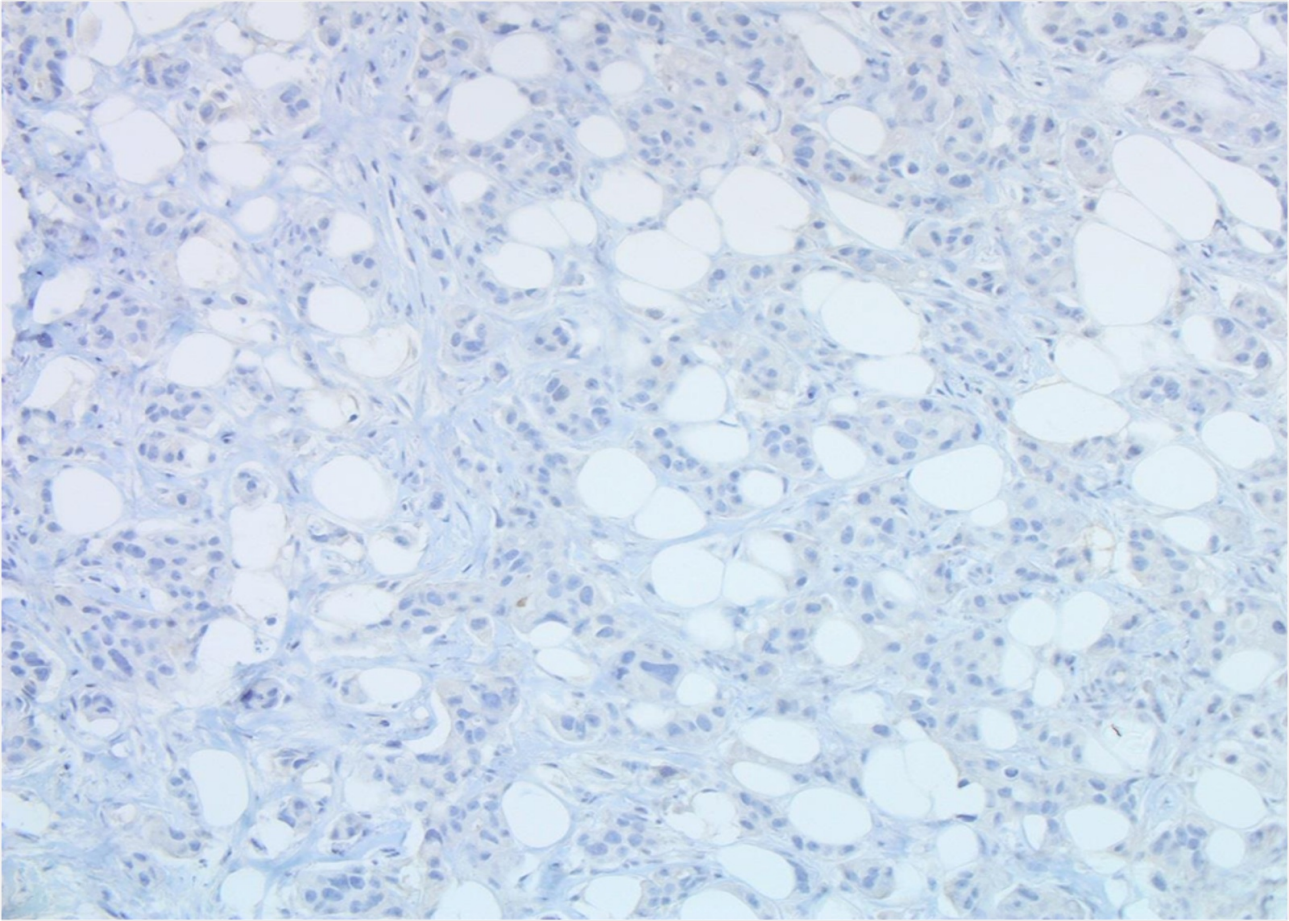
Patient 2: Tumor cells are ER-negative (40X)

A 73-year-old female was diagnosed with de novo metastatic HER2-positive/ER- and PR-negative breast cancer in July 2009. At presentation, she was found to have intra-thoracic lymphadenopathy and multiple lung nodules (Figure 9) in addition to a multifocal right-sided breast tumor (Figures 10-12) and enlarged axillary nodes.

**Figure 13:**
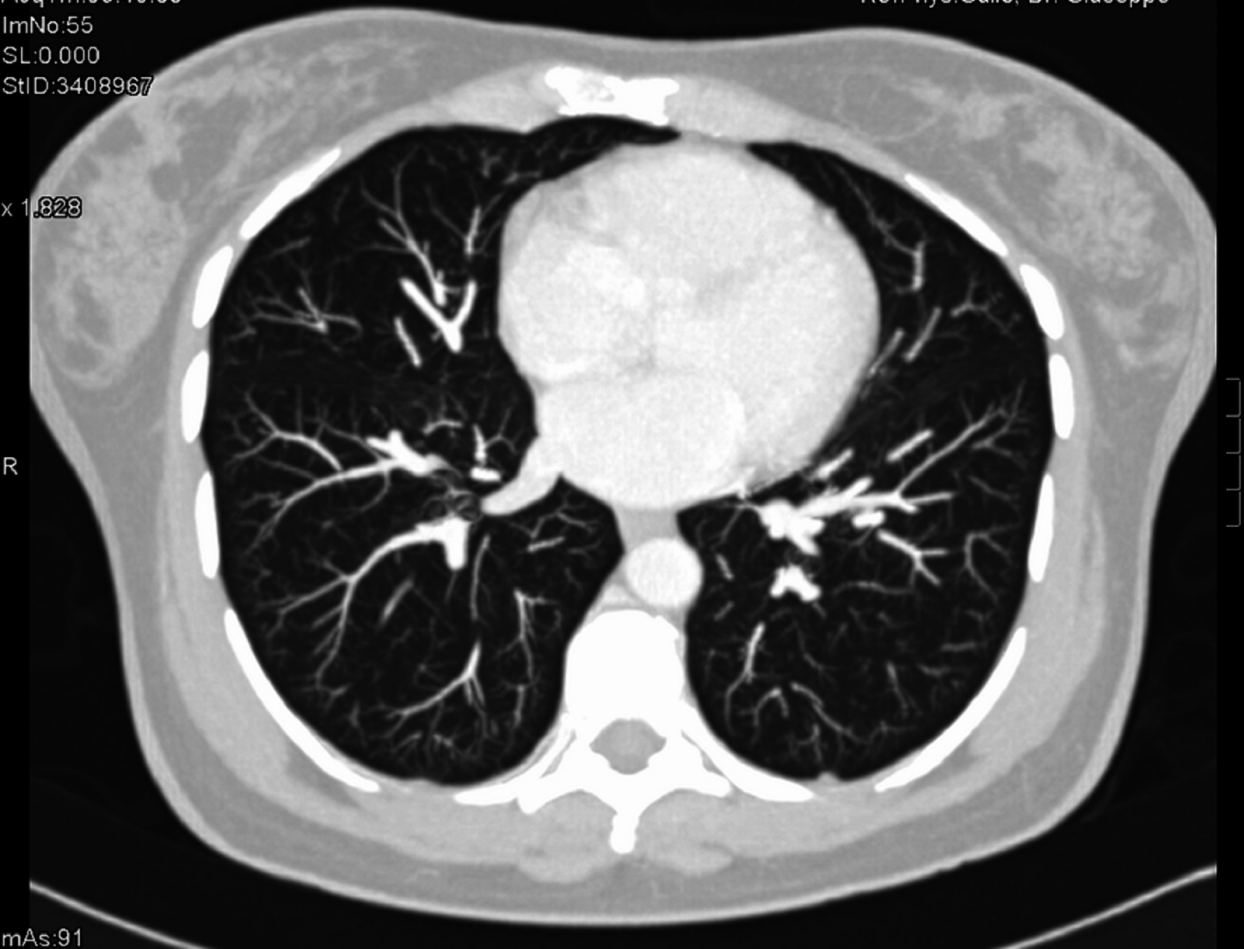
Patient 2: Computed tomography (CT) imaging demonstrates resolution of lung nodules post treatment

She was commenced on systemic chemotherapy and dual HER2-targeted agents in the form of three-weekly docetaxel, lapatinib, and trastuzumab as part of a clinical trial and achieved a CR (Figure 13). After cessation of docetaxel, she continued on a maintenance regime of lapatinib and trastuzumab. Lapatinib was permanently discontinued in July 2012 after approximately three years of treatment owing to gastrointestinal toxicity (persistent nausea, gastritis, and anorexia). She is currently maintained on three-weekly single-agent trastuzumab. Recent imaging reveals no evidence of metastatic disease nearly eight years following initial diagnosis.

Patient 3

**Figure 14:**
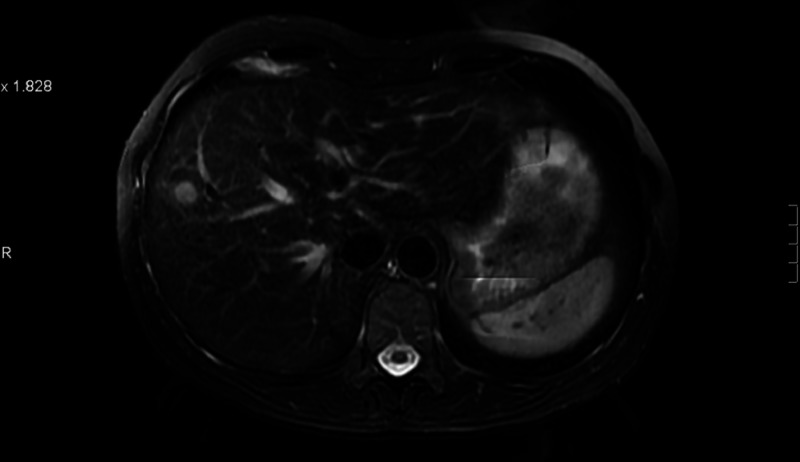
Patient 3: Magnetic resonance imaging (MRI) liver demonstrates single liver metastasis pre treatment

**Figure 15:**
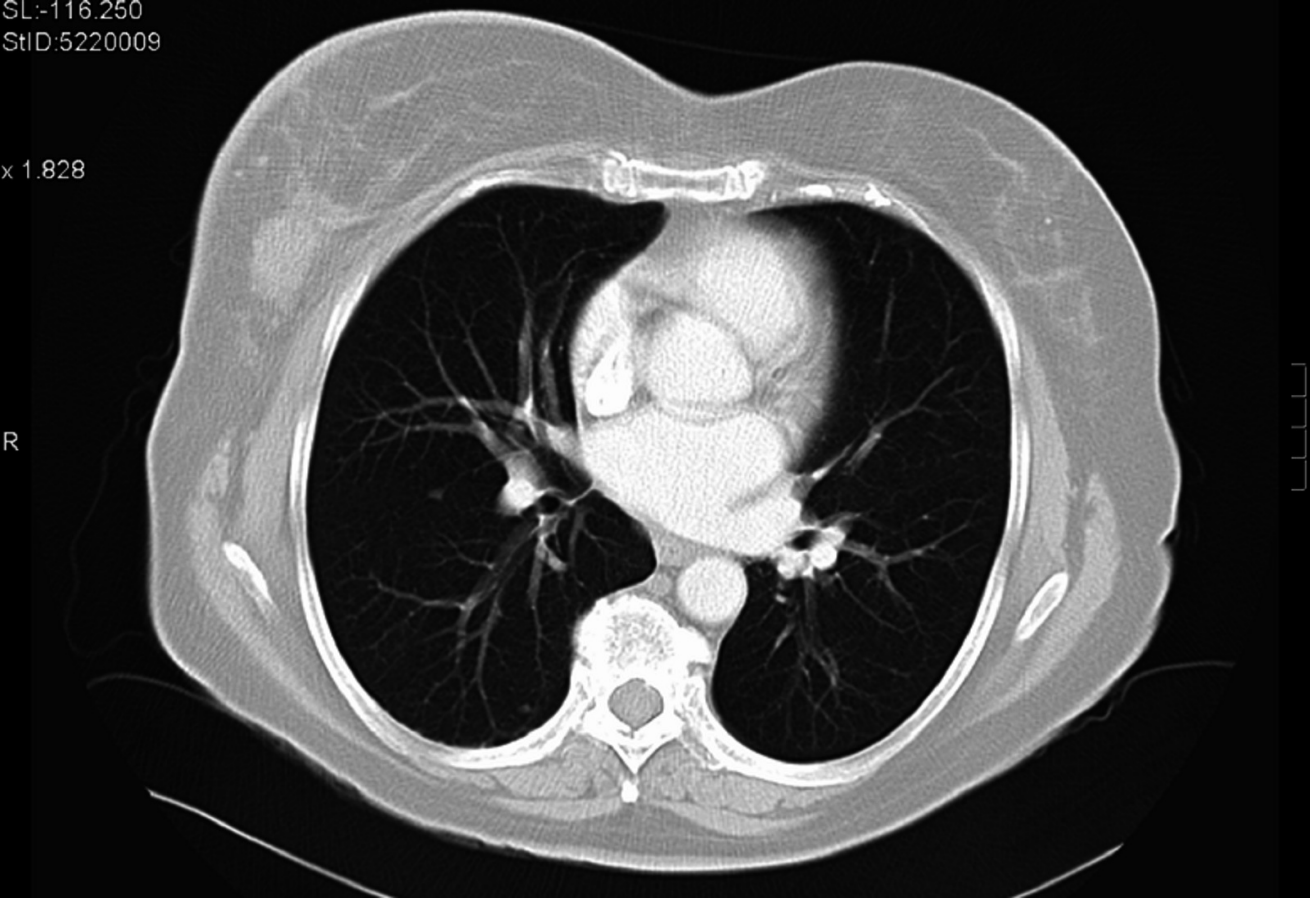
Patient 3: Computed tomography (CT) imaging reveals lung nodules pre treatment

A 61-year-old female presented with de novo metastatic HER2-positive/ER- and PR-negative breast cancer to liver and lung in March 2009 (Figures 14-15).

**Figure 16:**
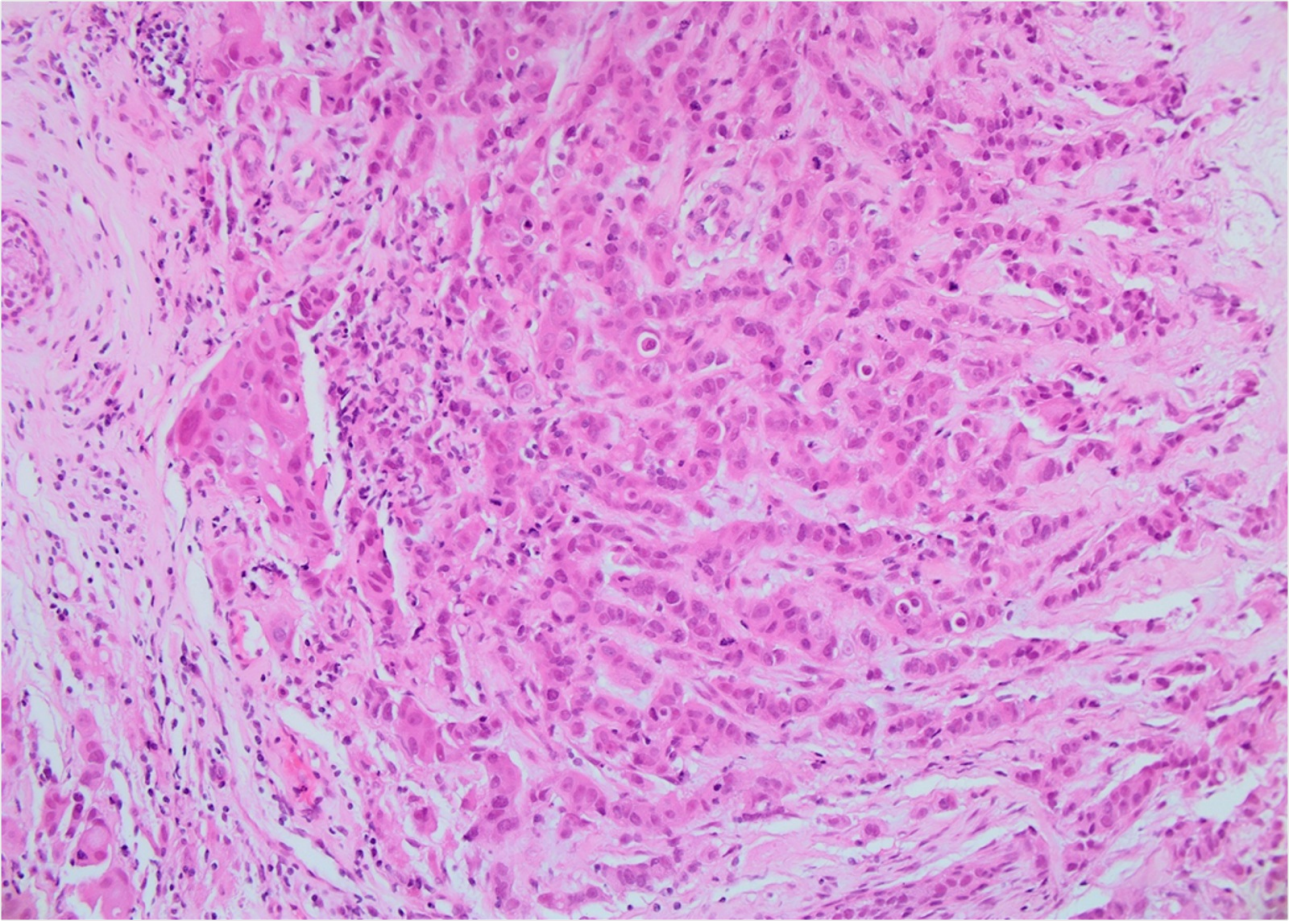
Patient 3: Biopsy of left breast tissue infiltrated by invasive ductal carcinoma (IDC) with features suggestive of Grade 3 pathology (20X)

**Figure 17:**
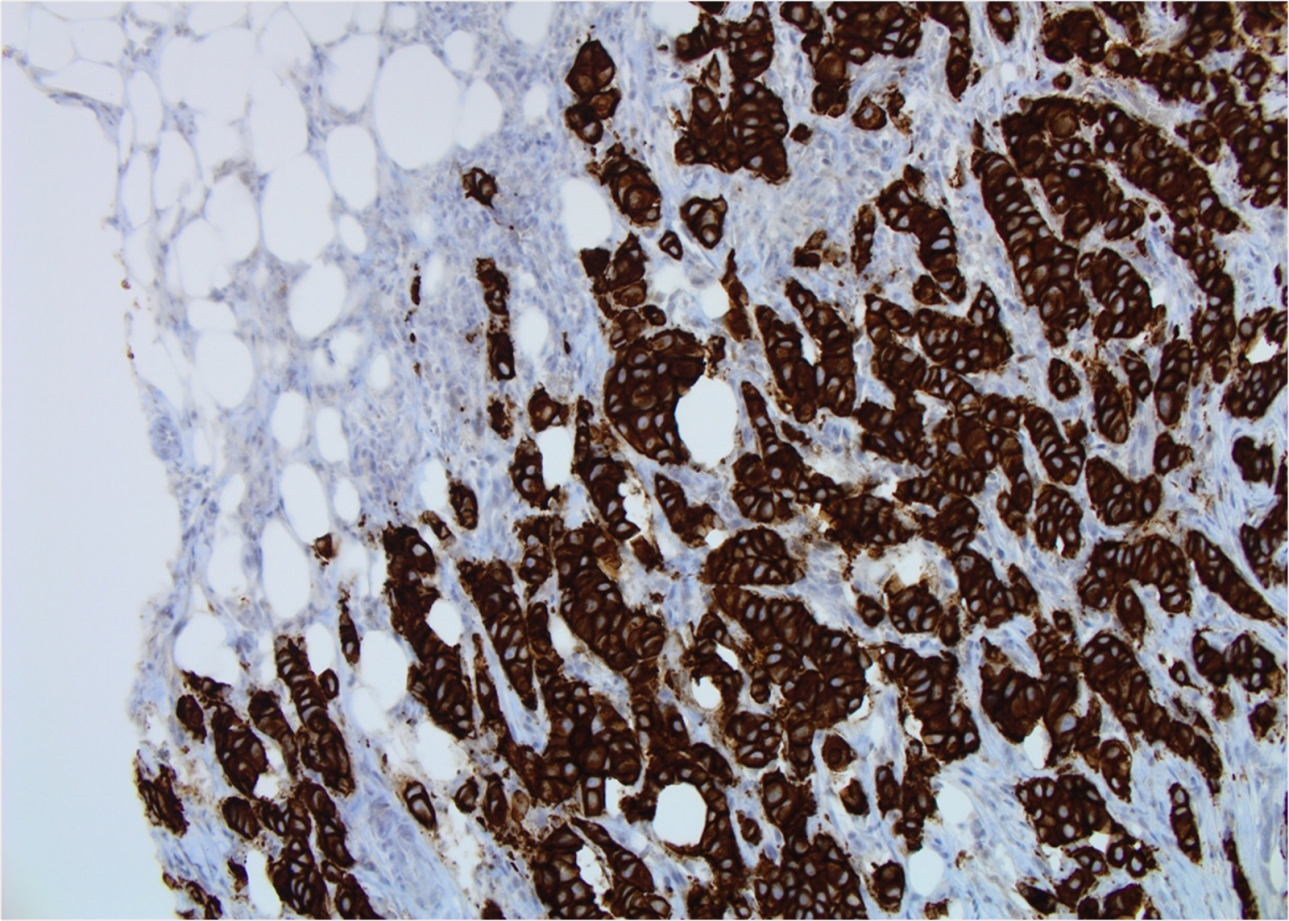
Patient 3: Tumor cells show strong membranous positivity for HER2 (score 3+). (20X)

**Figure 18:**
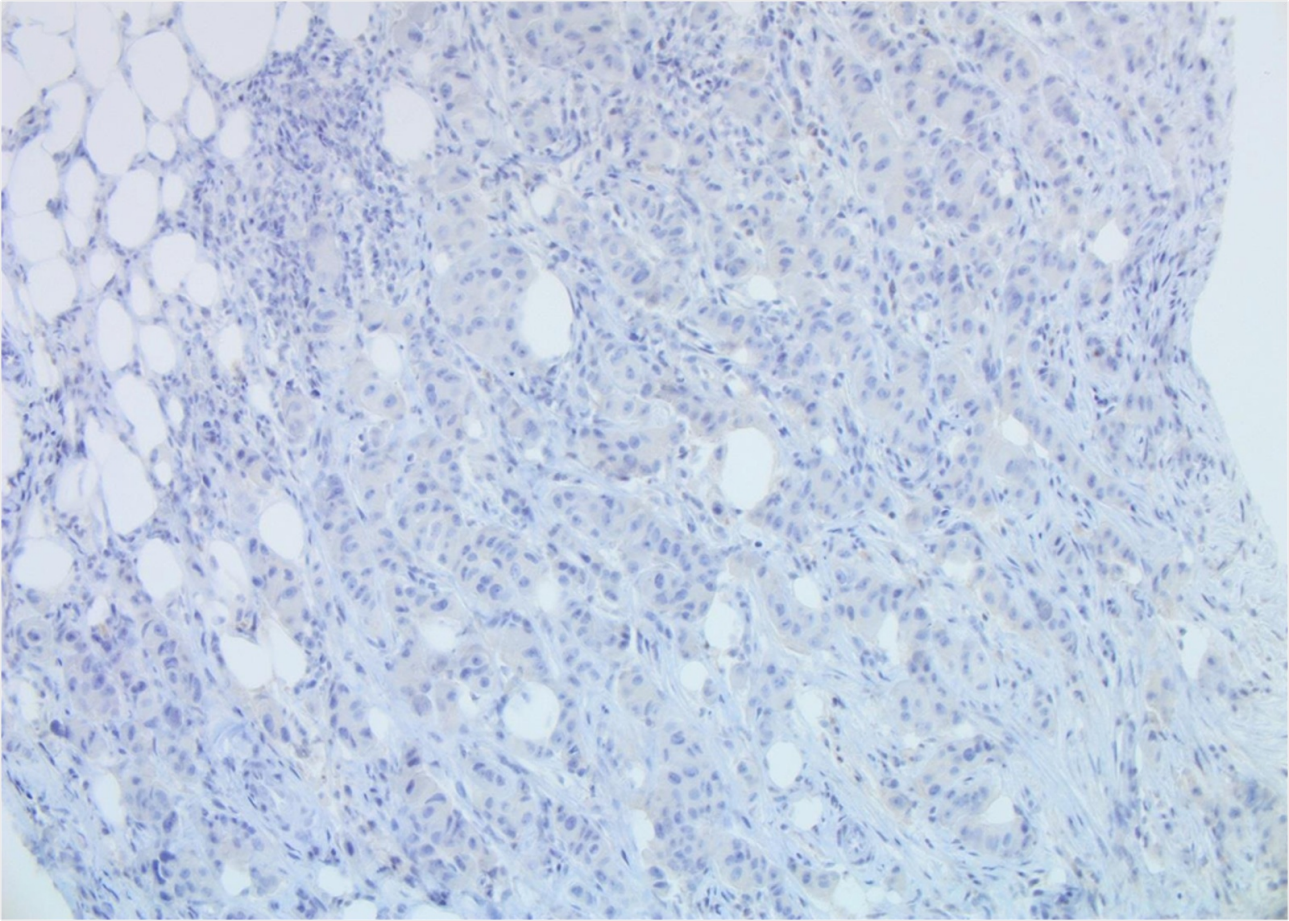
Patient 3: Tumor cells are ER-negative (40X)

**Figure 19:**
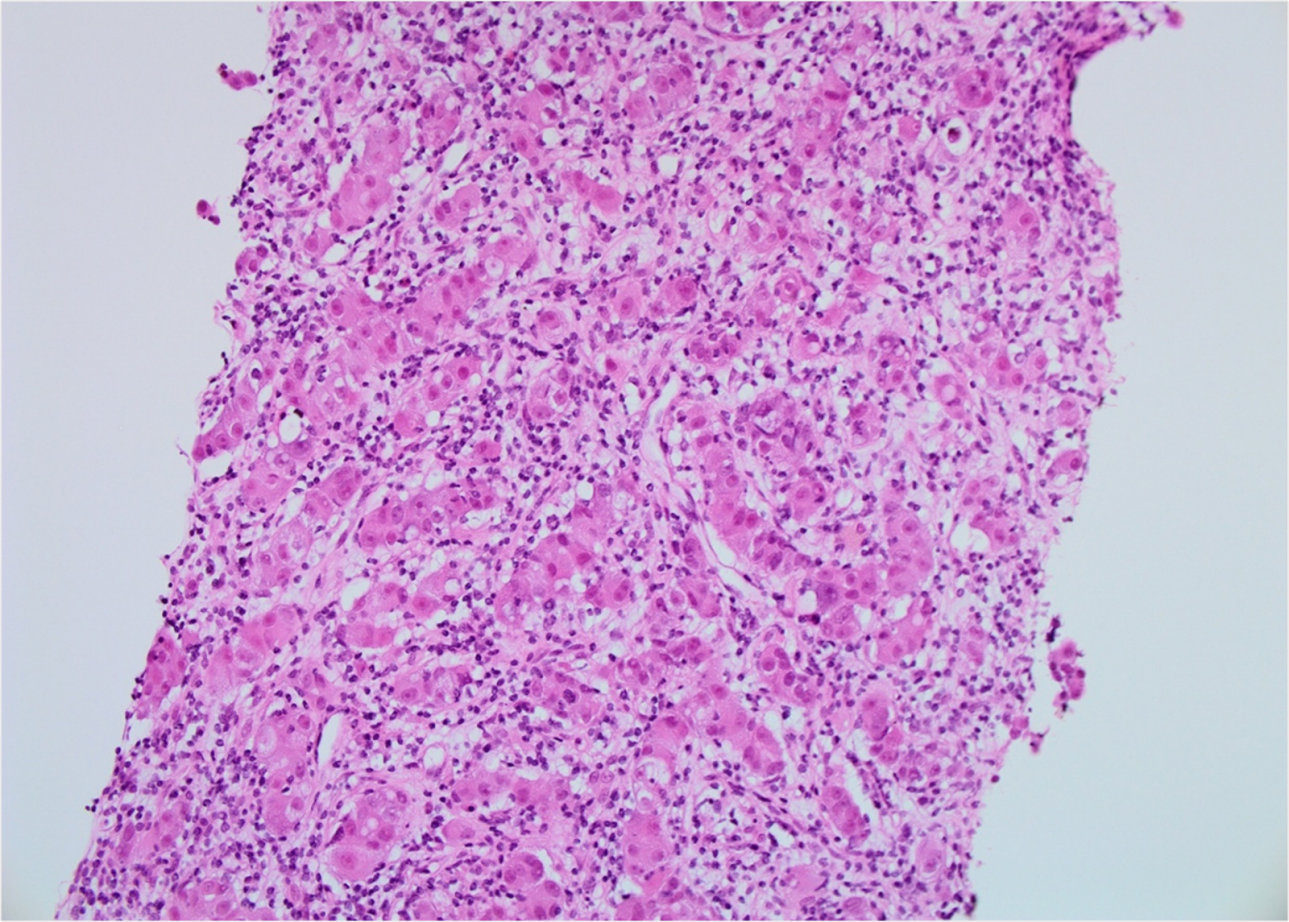
Patient 3: Biopsy of liver tissue infiltrated by metastatic ductal carcinoma (20X)

The diagnosis was confirmed on core biopsies of both breast primary tumor and liver metastasis (Figures 16-19).

**Figure 20:**
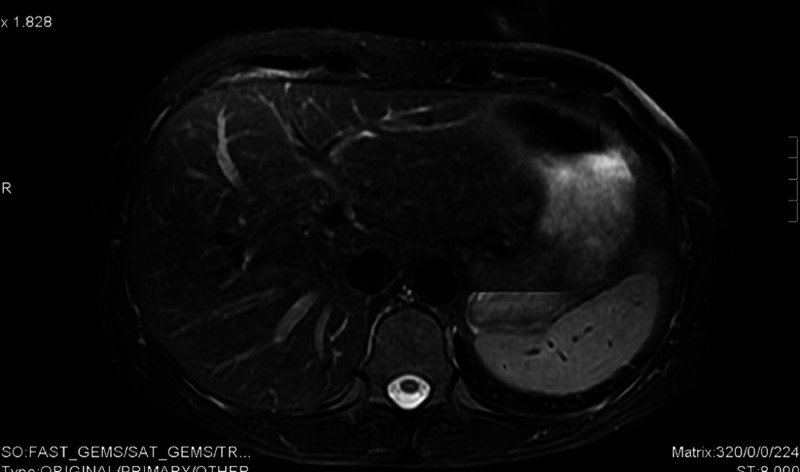
Patient 3: Magnetic resonance imaging (MRI) liver reveals resolution of liver metastasis post treatment

Her first-line treatment consisted of three-weekly docetaxel, lapatinib, and trastuzumab within a clinical trial. Following completion of cytotoxic chemotherapy, she was maintained on dual HER2-blockade. After about five months of such treatment, she achieved an excellent radiological response (Figure 20) and she was deemed suitable for definitive surgical intervention.

**Figure 21:**
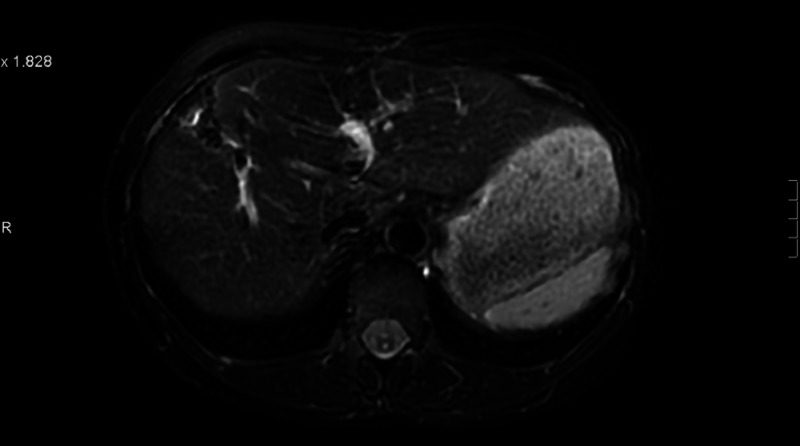
Patient 3: Magnetic resonance imaging (MRI) liver post resection demonstrates no evidence of recurrence

**Figure 22:**
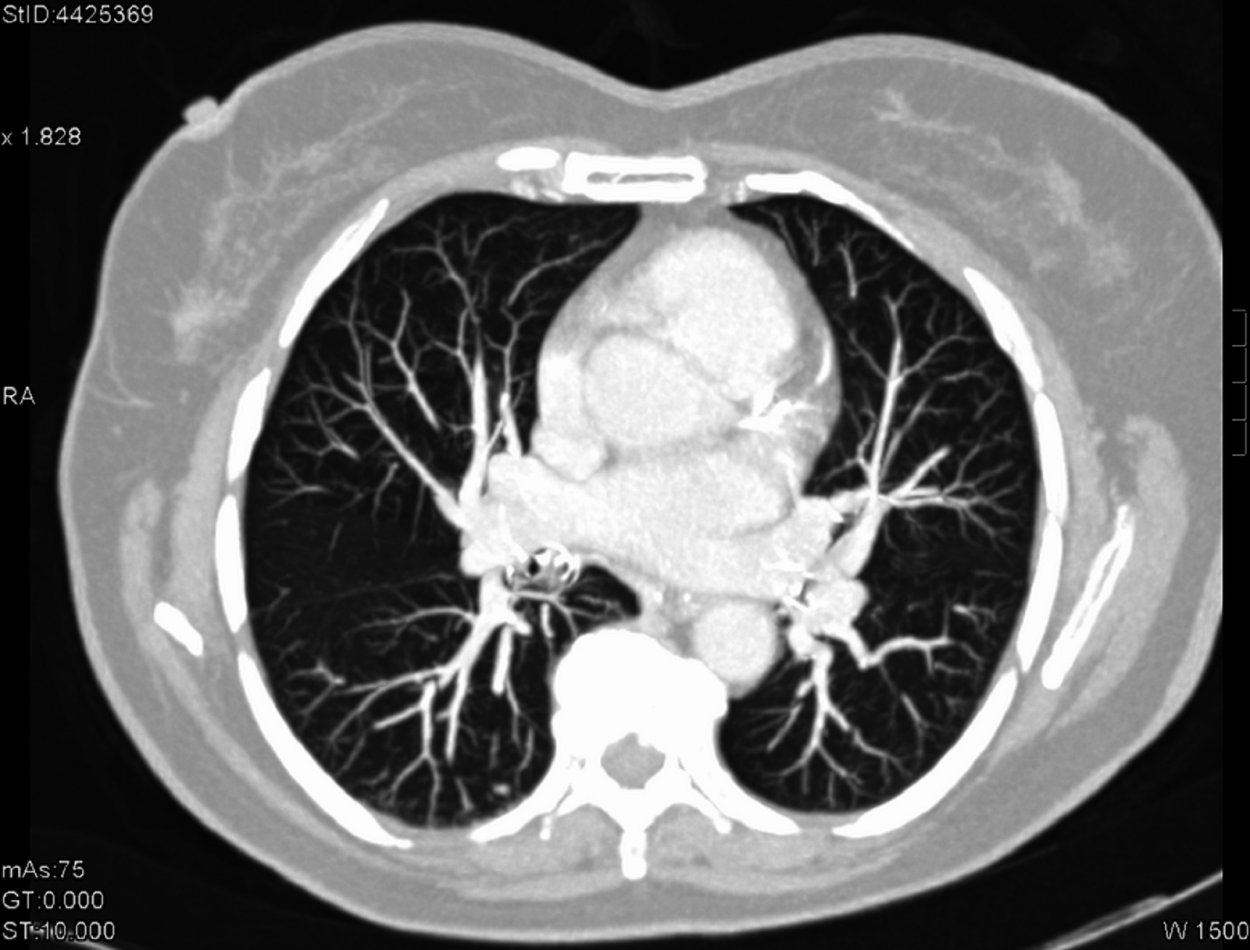
Patient 3: Computed tomography (CT) imaging reveals resolution of significant lung nodules and stability over eight years post initial treatment

A left wide local excision (WLE) and sentinel lymph node biopsy was performed in July 2009. This was followed by a liver resection of segment 5 and 8 in December 2009. Both breast and liver specimens were negative for invasive carcinoma, demonstrating complete pathological response to therapy. Computed tomography (CT) and magnetic resonance imaging (MRI) revealed no residual disease (Figure 21). Lapatinib was permanently discontinued in April 2014 to streamline therapy in light of her prolonged disease control. She remains disease free on single-agent maintenance trastuzumab alone, over eight years following original presentation (Figure 22).

## Discussion

Our case series illustrates that DCR is not anecdotal and is possible in a meaningful minority of patients with overtly metastatic HER2-positive breast cancer.

In the current molecular era of medical oncology, it is becoming increasingly imperative to identify the subset of patients who are likely to have a meaningful response to certain therapies. However, the identification of a biomarker or a panel of clinical and biological factors to predict DCR has been very elusive so far. In fact, studies to date have suggested that HER2-positive breast cancer is a rather heterogeneous disease with different clinicopathological factors and molecular signatures governing the likelihood of response or resistance to targeted therapy [6].

Our aforementioned retrospective series suggests that ER-negative disease is associated with a greater impact of trastuzumab in the metastatic setting [5]. A similar association was seen in studies assessing the efficacy of neoadjuvant therapy in the management of early HER2-positive breast cancer, namely the NOAH (NeOAdjuvant Herceptin) trial [7] and the NeoALTTO (Neoadjuvant Lapatinib and/or Trastuzumab Treatment Optimisation) trial [8]. However, prolonged phases of disease control and even DCRs can be seen sometimes in patients with strongly hormone-sensitive disease, thus indicating that ER or PR status is not an optimal predictive marker of DCR. A limited disease burden (i.e. one or two sites of distant metastases) as well as no previous exposure to trastuzumab have also been strongly linked with a higher likelihood of DCR. A prolonged phase of single-agent maintenance trastuzumab, together with hormonal manipulation in patients with hormone-sensitive disease, appears to play a meaningful role in preventing disease relapse and possibly conferring a long-term cure. Therefore, it is the practice within our institution to continue trastuzumab indefinitely after discontinuation of cytotoxic chemotherapy for patients who have achieved a CR [5, 9-10].

Data garnered from the Cancer Genome Atlas has confirmed there are five intrinsic subtypes among clinically confirmed HER2-positive tumors – HER2-enriched, luminal A, luminal B, basal-like, and normal-like [11]. Tumors of the HER2-enriched subtype (50% of clinical HER2-positive tumors) are thought to be associated with higher activation of the HER2/EGFR (epidermal growth factor receptor) signaling pathway. As a result, it would be hypothesized that they are more sensitive to HER2-directed therapies [12-13]. The phase II PAMELA (PAM50 HER2-enriched Phenotype as a Predictor of Response to Dual HER2 Blockade in HER2-positive Early Breast Cancer) trial further evaluated this theory in the neoadjuvant setting by comparing rates of pathological complete response to lapatinib and trastuzumab among the different molecular subtypes. This study found that 41% of patients with HER2-enriched subtype achieved pathological complete response (PCR) compared with 10% of patients with the non-HER2-enriched subtype [14].

A further study published recently in *Annals of Oncology* analyzed the influence of gene and pathway mutations on response to HER2-targeted therapies in the NeoALTTO trial [15]. Patients in this trial were randomized to lapatinib alone, trastuzumab alone, or the combination of both in combination with paclitaxel chemotherapy in the neoadjuvant setting for treatment of early stage HER2-positive breast cancer. A RhoA pathway mutation in a patient's tumor was associated with improved response to lapatinib alone and with combination HER2-inhibition therapy as well as with superior event-free and overall survival. The HER2 (ErbB2) receptor regulates microtubule activity in the cell by employing a complex containing RhoA. Mutations leading to inhibition of the RhoA pathway are therefore thought to result in reduced cell motility and invasion. Interestingly, no gene mutations or pathway alterations were associated with an improved PCR with trastuzumab alone. In contrast, a PIK3CA single gene mutation was associated with a lower response to therapy. Furthermore, 23 PIK3CA-associated pathway-level mutations were also associated with poorer response in all therapy arms [15]. The phosphatidylinositol 3-kinase (PI3K) pathway is frequently upregulated in breast cancer. A PIK3CA gene mutation is the most common mechanism of pathway enhancement and is observed in 30% of breast cancers. The PI3K protein is composed of a p85 and a p110 subunit. The PIK3CA gene encodes for the latter subunit. When mutated, this protein can acquire oncogenic properties, leading to activation of the Akt pathway independent of upstream signaling, which can result in increased cell proliferation, survival, and metastasis. Manipulation of this pathway is, therefore, an attractive treatment target and PI3K inhibitors are currently under investigation in clinical trials. They are also being investigated in combination with HER2-directed therapies given the association of PI3K pathway alterations and trastuzumab resistance [15-19].

Finally, the influence of tumor immunogeneity in provoking a response to HER2-directed therapies has not been fully explored yet. Full exploration may explain further why certain patients are more likely to respond to treatment. Trastuzumab is a monoclonal antibody; it has been theorized that at least part of its efficacy lies in its ability to augment the adaptive immune response in order to promote an anti-tumor effect via antibody-dependent cell-mediated cytotoxicity (ADCC) [6].

Despite these advances in our knowledge, it is unknown how to translate these findings into clinical practice. To help address this dilemma, we are conducting a comprehensive cytogenetic and molecular analysis of patients’ tumor specimens in order to identify the factors that would predict a complete disease response in patients with metastatic HER2-positive breast cancer.

## Conclusions

DCR is achievable in a numerically limited but clinically meaningful subset of patients with metastatic HER2-positive breast cancer, as our case series demonstrates. In view of the very prolonged duration of CRs, it can be speculated that HER2-positive breast cancer may be potentially curable in people who achieve exceptional responses. Several studies to date have demonstrated that DCR is more likely in patients with certain favorable clinicopathological and molecular factors such as de novo presentation (or no previous exposure to trastuzumab), ER-negative status, limited disease burden, and absence of deleterious gene or pathway mutations such as PIK3CA. Further research and algorithms are needed in order to confidently integrate these findings into clinical practice.
